# Non-viral gene coating modified IOL delivering PDGFR-α shRNA interferes with the fibrogenic process to prevent posterior capsular opacification

**DOI:** 10.1093/rb/rbad020

**Published:** 2023-03-08

**Authors:** Jiahao Wang, Yulin Hu, Yuemei Han, Qiuna Fang, Zhirong Chen, Yajia Wang, Peiyi Zhao, Hui Wang, Quankui Lin

**Affiliations:** Department of Biomaterials, National Engineering Research Center of Ophthalmology and Optometry, School of Biomedical Engineering, School of Ophthalmology and Optometry, Eye Hospital, Wenzhou Medical University, Wenzhou 325027, China; Department of Biomaterials, National Engineering Research Center of Ophthalmology and Optometry, School of Biomedical Engineering, School of Ophthalmology and Optometry, Eye Hospital, Wenzhou Medical University, Wenzhou 325027, China; Department of Biomaterials, National Engineering Research Center of Ophthalmology and Optometry, School of Biomedical Engineering, School of Ophthalmology and Optometry, Eye Hospital, Wenzhou Medical University, Wenzhou 325027, China; Department of Biomaterials, National Engineering Research Center of Ophthalmology and Optometry, School of Biomedical Engineering, School of Ophthalmology and Optometry, Eye Hospital, Wenzhou Medical University, Wenzhou 325027, China; Department of Biomaterials, National Engineering Research Center of Ophthalmology and Optometry, School of Biomedical Engineering, School of Ophthalmology and Optometry, Eye Hospital, Wenzhou Medical University, Wenzhou 325027, China; Department of Biomaterials, National Engineering Research Center of Ophthalmology and Optometry, School of Biomedical Engineering, School of Ophthalmology and Optometry, Eye Hospital, Wenzhou Medical University, Wenzhou 325027, China; Department of Biomaterials, National Engineering Research Center of Ophthalmology and Optometry, School of Biomedical Engineering, School of Ophthalmology and Optometry, Eye Hospital, Wenzhou Medical University, Wenzhou 325027, China; Department of Biomaterials, National Engineering Research Center of Ophthalmology and Optometry, School of Biomedical Engineering, School of Ophthalmology and Optometry, Eye Hospital, Wenzhou Medical University, Wenzhou 325027, China; Department of Biomaterials, National Engineering Research Center of Ophthalmology and Optometry, School of Biomedical Engineering, School of Ophthalmology and Optometry, Eye Hospital, Wenzhou Medical University, Wenzhou 325027, China

**Keywords:** intraocular lens, posterior capsule opacification, surface modification, non-viral gene delivery system, epithelial–mesenchymal transformation

## Abstract

Posterior capsule opacification (PCO), the most common complication after cataract surgery, is caused by the proliferation, migration and epithelial–mesenchymal transition (EMT) of residual lens epithelial cells in the capsule bag. Although the surface modification and drug loading of intraocular lens (IOLs) have been effective in preventing PCO to some extent, the intraocular safety of anti-proliferative drug application is still a major limitation in clinical application. In this study, we used non-viral gene delivery systems in combination with layer-by-layer (LBL) self-assembly technology, and the modified IOL could effectively prevent the development of PCO by interfering with the EMT process mediated by the platelet-derived growth factor receptor-α (PDGFR-α). Herein, the gene fragments were wrapped by electrostatic conjugation using polyethyleneimine-graft-poly(ethylene glycol) to form gene complexes. Gene complexes were characterized by dynamic light scattering, transmission electron microscopy (TEM) and agarose gel electrophoresis, and evaluated for storage and serum stability. The layer assembly behavior of the IOL surface, changes in optical properties and the release behavior of the gene complexes were characterized using quartz crystal microbalance, UV–vis, contact angle and TEM. *In vitro* experiments showed that the IOL coating has good bio-compatibility and can achieve the corresponding transfection effect, and the released gene complexes exhibited excellent cell internalization and lysosomal escape behaviors, as well as effective inhibition of PDGFR-α expression and its mediated EMT process. The early PCO prevention effect and bio-compatibility evaluation of the modified IOL *in vivo* were evaluated by implantation into animal eyes. This study provides a new strategy for the development of surface modifications of small nucleic acid drugs and non-toxic EMT interference therapies for PCO.

## Graphical Abstract

**Figure:**
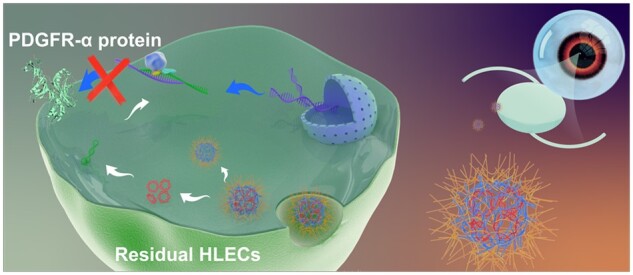

## Introduction

As the world’s leading blindness-causing and vision-impairing eye disease, cataracts are a constant threat to people’s vision and quality of life [1–3]. Surgical extraction is the only effective intervention for cataracts, and the most common procedure is phacoemulsification combined with intraocular lens (IOLs) implantation [4]. However, the most common postoperative complication associated with this procedure is posterior capsule opacification (PCO) [5, 6]. The incidence of PCO at 3 years postoperatively has been reported to be 20–40% in adults and nearly 100% in infants and children [7, 8]. To resolve this condition, the patient will need to undergo another posterior capsule dissection of the Neodymium-doped Yttrium Aluminum Garnet (Nd: YAG) laser lens for treatment [9]. The psychological impact of secondary surgery aside, infant patients are not only unable to cooperate with treatment, but can also suffer from a host of new complications such as iris hemorrhage, elevated intraocular pressure, macular cystoid edema and retinal detachment [10, 11]. Therefore, effective prevention of PCO to avoid secondary surgical injuries remains a major research priority in this field.

From the perspective of pathogenesis, PCO is mainly due to the continuous proliferation, migration and epithelial–mesenchymal transition (EMT) of lens epithelial cell (LECs) remaining in the capsular bag in the pore between the lens and the capsular membrane [12–14]. EMT as the body’s repair process in the eye leads to a pathological fibrotic process that destroys the hyaline medium [15, 16]. During the development of PCO, the EMT of LECs has increased the turbidity of the capsule after the IOL, especially the optical area, causing secondary blindness in patients.

Platelet-derived growth factor receptor-α (PDGFR-α) is associated with EMT in multiple diseases, and its stem cell and mesenchymal phenotype induced by activation of downstream MAPK/ERK, PI3K/AKT and STAT3 pathways have been demonstrated [17–19]. In addition, PDGFR-α expression triggers the migratory, invasive properties of the cells, transforming the 3D structure of the cells into a highly branched morphology [19]. Therefore, the inhibition of PDGFR-α protein expression by small nucleic acid drugs and thus interfering with the EMT process in LECs may be a new strategy for the prevention of PCO.

In recent years, surface modification of materials has received increasing attention from researchers due to its achievability and effectiveness in improving the biocompatibility of medical implant materials [20–22]. In addition to being an important refractive medium in the eye, IOLs can also serve as a drug delivery platform [23–25]. Various material surface modification techniques are used to build a multifunctional drug coating on the surface of the IOL, allowing it to carry the drug into the eye after implantation to inhibit PCO [26–28]. Currently, drug-loaded IOLs have been successfully developed and extensively studied [29, 30]. Layer-by-layer (LBL) technology has been widely used in biomedical applications, especially for drug delivery, protein delivery, tissue engineering and wound healing, because of its simplicity, efficiency, reproducibility and flexibility [31–33]. The thickness of multilayers assembled on the various types of surfaces can be well controlled at the nanoscale, which brings many advantages, such as higher surface area or specific side effects, leading to different pharmacokinetics or biodistribution for the delivery of drugs and bioactive substances [34–36].

The greatest challenges in gene therapy are low immunogenicity, good tolerability and effective delivery vectors [37–40]. Polycationic materials can be a promising vehicle for gene therapy of diseases due to their simple accessibility and unique immune escape capability [41, 42]. Polyethyleneimine (PEI) is one of the most successful polycationic carriers, and to improve its performance, polyethylene glycol (PEG) monoblock copolymers were used instead of PEI to increase biosafety [41, 43, 44]. In contrast, PEI–g–PEG non-viral gene vectors have been demonstrated in our previous experiments to have high uptake and low toxicity [45], and have achieved good results in delivering model protein particles. Thus, PEI–g–PEG can be used as a gene delivery tool to deliver PDGFR-α interfering fragments.

**Scheme 1.**
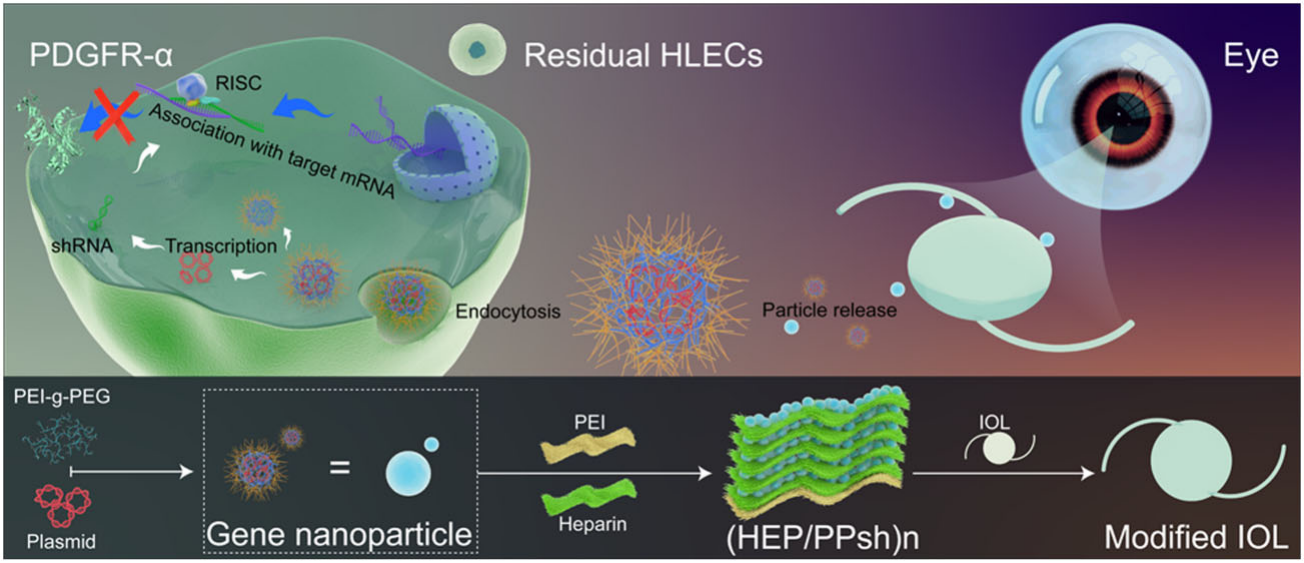
Schematic diagram of LBL construction of bioactive coating on IOL surface and gene delivery of PDGFR-α shRNA plasmid to interfere with EMT process for prevention of PCO.

Here, in this study, PEI–g–PEG/PDGFR-α shRNA gene nanoparticles were prepared by electrostatic conjugation method and combined with LBL assembly technology to design an IOL covered with genetically active multilayers as a way to achieve local release of therapeutic nanoparticles in the capsule and IOL interstitial space. The aim is to interfere with the migration, differentiation and proliferation of LECs from the perspective of EMT pathogenesis to achieve safe and effective PCO prevention (Scheme 1).

## Materials and methods

### Reagents and antibodies

Branched PEI with a molecular weight of 25 000 Da, bovine serum albumin and fluorescein isothiocyanate (FITC) were purchased from Sigma-Aldrich. Poly(ethylene imine)-graft-poly(ethylene glycol) (PEI–g–PEG, PEG Mn = 2000 Da, PEI Mn = 25 000 Da) was provided by Ruixi Bio. Fetal bovine serum (FBS), DMEM/F12 (1:1) cell culture medium, 0.05% trypsin–EDTA, penicillin–streptomycin solution and other cell-related culture reagents were purchased from Gibco. Phosphate-buffered saline (PBS) was purchased from Boster Biotechnology. Salmon sperm DNA and heparin sodium salt were purchased from Aladdin. Cell Counting Kit-8 (CCK-8), Hoechst 33342, Calcein/PI Cell Viability/Cytotoxicity Assay Kit, 4% Paraformaldehyde Fixative, Lyso-Tracker Red (Lysosomal Red Fluorescent Probe), DiI (Cell Membrane Red Fluorescent Probe), DAPI Staining Solution, Plasmid Maxi Preparation Kit for All Purpose, Nucleic Acid Electrophoresis and Recovery Mini-Package, Antifade Mounting Medium with DAPI and BeyoClick™ EdU Cell Proliferation Kit with Alexa Fluor 488 were purchased from Beyotime Biotechnology Co.

PDGFR-α shRNA plasmid DNA was purchased from Tsingke Biotechnology Co. PDGFR-α and E-cadherin antibodies for immunofluorescence (IF) were purchased from Santa Cruz and Immunoway. All secondary antibodies were from Santa Cruz. *In vitro* characterization and experiments with polyethylene terephthalate (PET) as a material. Foldable hydrophobic acrylic IOL provided by 66 Vision Technologies (Suzhou, China).

### Cell culture, plasmid extraction and animals

Human LECs (HLECs) were proliferated using a complete medium containing DMEM/F12, 10% FBS and penicillin–streptomycin solution. Cells were cultured at 5% CO_2_, 37°C. The medium was changed every 2–3 days. In other relevant experiments, cells were trypsinized and seeded in 96- or 24-well plates [46].

The reporter plasmid DNA encoding enhanced red fluorescent protein (RFP-pCAGGS) was propagated in *Escherichia coli* DH5α and extracted and purified using the Plasmid Maxi Preparation Kit [47]. The strain was kindly provided by Prof. Jieguang Chen (Wenzhou Medical University). PDGFR-α shRNA plasmid DNA (PTSB-SH-mCherry-2A-NEO) was also purified using the same method as described above. The concentration of DNA was subsequently determined by measuring the UV absorbance at 260 and 280 nm using a UV–vis spectrophotometer (DS-11, Denovix, USA). The purified DNA was stored at −20°C.

New Zealand White rabbits weighing 2.5–3 kg were purchased from the Animal Experiment Center of Wenzhou Medical University. Animal experiments were conducted in accordance with the relevant regulations of the Animal Ethics Committee of Wenzhou Medical University and the approved experimental protocol.

### Preparation and characterization of gene complexes

To facilitate the experiments, Salmon sperm DNA was used as a template reporter plasmid for subsequent characterization experiments [48]. PEI–g–PEG/DNA (PPD) complex samples were obtained using an electrostatic conjugation method and PEI/DNA (PD) made by the same method was used as a control for related experiments. As an example of PPD, briefly, polycationic PEI–g–PEG was dissolved with salmon sperm DNA using ultrapure water and the DNA solution was dropped into PEI–g–PEG solution with different N/P (molar ratio of the amino group of PEI–g–PEG to phosphorus element of DNA). The solution was vortexed for 30 s and then left for 30 min at room temperature to form the PPD complex solution.

The size distribution of PD and PPD prepared above and the zeta potential of PPD (N/P = 10) were analyzed by dynamic light scattering (DLS, Malvern Instrument Ltd, Malvern, UK) [49]. To assess the ability of PEI–g–PEG to encapsulate plasmid DNA, agarose gel electrophoresis was used to detect unconjugated nucleic acid fragments in PPD solutions under different N/P conditions. Briefly, 10 μl of PPD suspensions with different N/P ratios (1, 2, 5, 10 and 20) were run on a 2.5% (W/V) agarose gel containing 0.5× TBE NA-Red buffer. The results were subsequently analyzed using a digital imaging system [50] (Bio-Rad, GelDoc XR+, USA). Surface morphology and 3D structure of PPD (N/P = 10) complexes were observed using transmission electron microscopy (TEM, FEI Talos F200, America).

### Gene complex stability assay

To test the storage stability of the gene complexes, the prepared PPD (N/P = 10) solution was stored at rest in a 4°C refrigerator and its particle size potential was tested continuously for 7 days [51]. Similarly, the prepared PPD (N/P = 10) solution was mixed with a 20% concentration of BSA in equal volume, so that the final concentration of BSA in the solution was 10% (commonly used culture conditions for cell culture), to simulate the serum albumin condition *in vitro*. The samples were incubated continuously for 7 days, and the same volume was taken for gel electrophoresis experiments. The serum stability of PPD was assessed by observing whether BSA disrupts the ability of PPD to associate and causes leakage of nucleic acids.

### Preparation and characterization of the surface coating of IOLs

It has been proved by the aforementioned experiments that PPD nanoparticles are positively charged and can be immobilized on the material surface with negatively charged heparin by electrostatic interactions. The specific process for the fabrication of (HEP/PPD)*n* multilayer films on IOL is shown in Scheme 1. The materials were first sonicated in anhydrous ethanol and deionized water sequentially. Afterward, they were immersed in PEI solution (3 mg/ml) overnight, and after washing using deionized water, they were dried with nitrogen at room temperature to produce a positively charged aminated surface. Subsequently, the treated materials were immersed in heparin solution and PPD solution for 20 min each, rinsed three times with deionized water and dried under nitrogen gas. The above steps were repeated *n* times to obtain (HEP/PPD)*n* multilayer membrane [29].

The LBL self-assembly process on the material surface was characterized using a quartz crystal microbalance (QCM, QSense Explorer, Sweden) [52, 53]. Briefly, gold-coated quartz wafers are first functionalized by amination in PEI solution. The electrolytic cell is then washed with PBS until baseline equilibrium. The heparin solution was replaced after water rinsing until the frequency stabilized, and when smooth, the PPD solution was added. The above process was repeated five times, and the changes of surface frequency and depletion were recorded, and the changes of thickness and quality of each layer were also analyzed by this process. A UV spectrophotometer (UV-1780, Shimadzu, Japan) was used to measure the absorbance at 260 nm of the singular layer to characterize the success of the assembly process, and a water contact angle (WCA, OCA 15EC DataPhysics, Germany) was used to measure the change in surface wettability of each coating, and the values were photographed and recorded [54, 55].

### Characterization of the release process of gene complexes

To demonstrate the smooth release of gene nanoparticles from the multilayer membrane on the material surface, the modified material was placed in PBS buffer and the change of absorbance at 260 nm of the release solution was detected using a UV spectrophotometer. Similarly, a quartz wafer with a (HEP/PPD)5 multilayer film attached was rinsed at high frequency with the aid of QCM and the surface frequency change was recorded. In addition, the presence of spherical material in the rinse solution was observed using TEM as a way to identify whether the gene complex could be released.

### Inspection of optical properties of modified IOLs

To investigate the effect of this gene complex coating on the optical quality of IOL, (HEP/PPD)5 multilayer films were prepared on the surface of IOL according to the coating preparation method described above and the unmodified IOL was thought as a blank control. The clarity of this modified IOL in the test pattern was observed using an optical resolution plate (USAF1951) and photographed and recorded using a stereoscopic microscope (SMZ 18, Nikon, Japan). In addition, UV–vis was used to measure the change in transmittance before and after the modification to observe the effect of the coating on the IOL transmission performance.

### *In vitro* biocompatibility evaluation

The primary evaluation criterion for non-viral gene vectors as transfection reagents is whether they are safe for HLECs. HLECs were cultured in 96-well plates with 5 × 10^3^ cells per well and complete medium was added. When the cells were plastered, gradient concentrations of PEI–g–PEG and PEI polymer solution were added to give final concentrations of 2, 4, 6, 8 and 10 μg/ml, respectively. When the cells were incubated for 48 h, the original medium was replaced with fresh medium and 10 μl of CCK-8 reagent was added to each well and incubated at 37°C for 2 h. After incubation, the absorbance values were measured using a microplate reader (SpectraMax 190, Molecular Devices, USA) to detect the absorbance values at 450 nm [56]. This was used to compare the toxicity and safety of different concentrations of PEI–g–PEG and PEI [45].

In addition, PET resin (PET) sheets were used as the substrate for the coating modification [56, 57]. After sterilization by UV irradiation for 2 h, the (HEP/PPD)*n* multilayer films were prepared in the ultra-clean table according to the coating preparation method described above and cut into small discs with a diameter of 6 mm. The small discs of each group were placed in the wells of 96-well culture plates in advance, and the plates were seeded and assayed according to the above method to analyze the effect of multilayer membranes on cells. To further characterize the multilayer membrane toxicity after gene nano complex modification, cells were stained after 48 h of incubation using the Calcein/PI cell viability/cytotoxicity assay kit [49]. Cells were then placed in an inverted fluorescence microscope (DMi8, Leica, Germany) and fluorescence images were recorded.

### Analysis of cellular uptake capacity

To facilitate observation of cell uptake, FITC-labeled PEI–g–PEG was used to prepare the complex P^FITC^PDNA [58]. HLECs were cultured in 24-well plates (wells were spiked in advance with cell culture slide) at a density of 4 × 10^4^ cells per well. When the cells were in good apposition, the prepared P^FITC^PDNA was added dropwise into the well plate and 4% paraformaldehyde was added for cell fixation for 30 min after incubation for 10 min, 30 min, 1 h, 2 h, 4 h and 6 h, respectively. After the cell membrane was stained by DiI and the floating color was removed by PBS, the antifade mounting medium with DAPI was added dropwise and the film was sealed. Finally, images of each fluorescence channel were taken with the help of a confocal laser scanning microscope (LSM 880, Zeiss, Germany).

In addition to this, the cell uptake efficiency was quantified using a flow cytometer (BD FACS Calibur, Becton, Dickinson and Company, USA). Cell culture and staining steps were performed as above, followed by cells filtered through 300 mesh, washed with PBS and top-sampled for detection.

### Analysis of lysosomal escape capacity

After internalization of the gene nanoparticles in the cytoplasm, the step of smooth escape from lysosomal degradation of nucleic acid fragments is crucial for the subsequent function of the plasmid DNA [59]. To observe this process, P^FITC^PDNA was also used and lysosomes were stained and labeled to observe the co-localization effect to laterally characterize the lysosomal escape behavior. Briefly, again as in cell uptake experiments cell seeding plates were performed, samples were added, cells were fixed at 30 min, 1 h, 2 h, 4 h, 6 h, 8 h and 24 h time points, rinsed and then stained for lysosomes using Lyso-Tracker Red (LT-Red). After washing away the floating colors, the slices were sealed, and other steps were performed as before. Fluorescence signals of each channel were acquired under a confocal laser scanning microscope. The P^FITC^PDNA green signal was observed under the same parameters as the LT-Red red signal for co-localization analysis.

### Transfection capacity exploration

PEI–g–PEG transfection complexes with N/P ratios of 10 and 20 were prepared according to the same method described above. The plasmid DNA containing the gene encoding red fluorescent protein was encapsulated, which was named PEI–g–PEG/RFP (PP/RFP), and the modified multilayer membrane was named (HEP/PPRFP)*n*. HLECs cells were inoculated in 24-well plates at 4 × 10^4^ and replaced with medium containing PP/RFP when the cells were in a good adherent growth state. After transfection for 48 h, the red fluorescence expression was observed under an inverted fluorescence microscope, while the microstructure was recorded under a confocal laser scanning microscope after DAPI-impregnated cell nuclei [47].

Meanwhile, to quantify the transfection efficiency of each ratio of PP/RFP, the transfected cells were recorded under flow cytometry (FCM) for the red fluorescence signal and statistically analyzed. Afterward, PET sheets containing (HEP/PPRFP)*n* multilayer membranes were covered over the well-adhered cells for *in vitro* mock IOL transfection. Forty-eight hours later, the cells were observed and recorded under a fluorescence microscope.

### IF analysis

Nano complex PEI–g–PEG/PDGFR-α shRNA was prepared, in which four PDGFR-α shRNAs were selected for screening (named as PPshn), whose coding sequence is as follows:

**Table:** 

shRNA1	CCGGGATGATCTGCAAGCATATTAACTCGAGTTAATAYGCTTGCAGATCATCTTTTTT
shRNA2	CCGGGCCAGCAATCTCTCAAATATTCTCGAGAATATTTGAGACATTGCTGGCTTTTTT
shRNA3	CCGGCGTTCAAGACCAGCGAGTTTACTCGAGTAAACTCGCTGGTCTTGAACGTTTTTT
shRNA4	CCGGAGTGGCCATTACACCATTATACTCGAGTATAATGGTGTAATGCCACTTTTTTT

After treatment with PPshn transfection for 48 h, cells were fixed with 4% pre-cooled paraformaldehyde for 30 min. After subsequent washing with PBS, permeabilization with 0.3% Triton X-100 and closure with 5% BSA, the cells were incubated with primary antibodies (Fibronectin and PDGFR-α) overnight at 4°C. After washing three times with PBS, samples were incubated with secondary antibodies for 1.5 h at room temperature [60]. The antifade mounting medium with DAPI was added and the images of several individual fluorescence channels were recorded with a confocal laser scanning microscope.

### Cell scratch assay

To investigate the effect of interference with PDGFR-α protein expression on cell migration, cell scratch experiments were performed [61]. Cells were first cultured in 24-well plates containing 500 μl of complete medium per well at a density of 4 × 10^4^ cells/well. After 24 h of culture, a cell scratch was made with a sterile gun tip, and then free cells were removed with PBS and divided into five groups. Four media containing PPsh gene complexes were added and normal complete media served as the control group. Subsequently, the cells were continuously observed for 84 h and the cells in the same area were photographed and observed at different time points.

### *In vivo* PCO prevention effect study

To evaluate the *in vivo* therapeutic effect of (HEP/PPsh2)5-modified IOLs, an animal model of PCO was established for 2.5–3 kg Japanese white rabbits undergoing phacoemulsification combined with IOL implantation [54–56]. Rabbits were treated, monitored and evaluated according to the Society for Research in Vision and Ophthalmology guidelines. Animal experiments were approved by the Experimental Animal Ethics Committee of Wenzhou Medical University. The body weight and intraocular pressure of the rabbits were routinely checked before the operation. The congenital eye diseases and mental disorders of the rabbits were ruled out by slit lamp examination and routine observation, respectively. The right eye was selected as the experimental eye for all surgeries, and 10 rabbits were divided into two groups and implanted with unmodified IOL (control group) and (HEP/PPsh2)5-modified IOL (experimental group), respectively. Thanks to Dr Han for her great contribution to animal surgery.

Post-operatively, after pupil dilation, the development of PCO was examined using a slit lamp for a period of time after surgery, while observing acute ocular inflammation, including corneal edema, anterior chamber exudation and inflammation. At the end of the experiment, the rabbits were humanely executed and the bilateral eyeballs were removed, and the lens capsular were carefully separated and stained with hematoxylin–eosin (HE) stain. The extent of PCO was further assessed by observing the morphology of the ocular tissue and the thickness of the posterior capsule hyperplasia. The corneas, iris, anterior chamber angle and retina of rabbit eyes were similarly isolated and pathologically stained to observe the morphology of each tissue to assess their biocompatibility *in vivo*.

## Results and discussion

### Preparation and characterization of gene complexes

**Figure 1.**
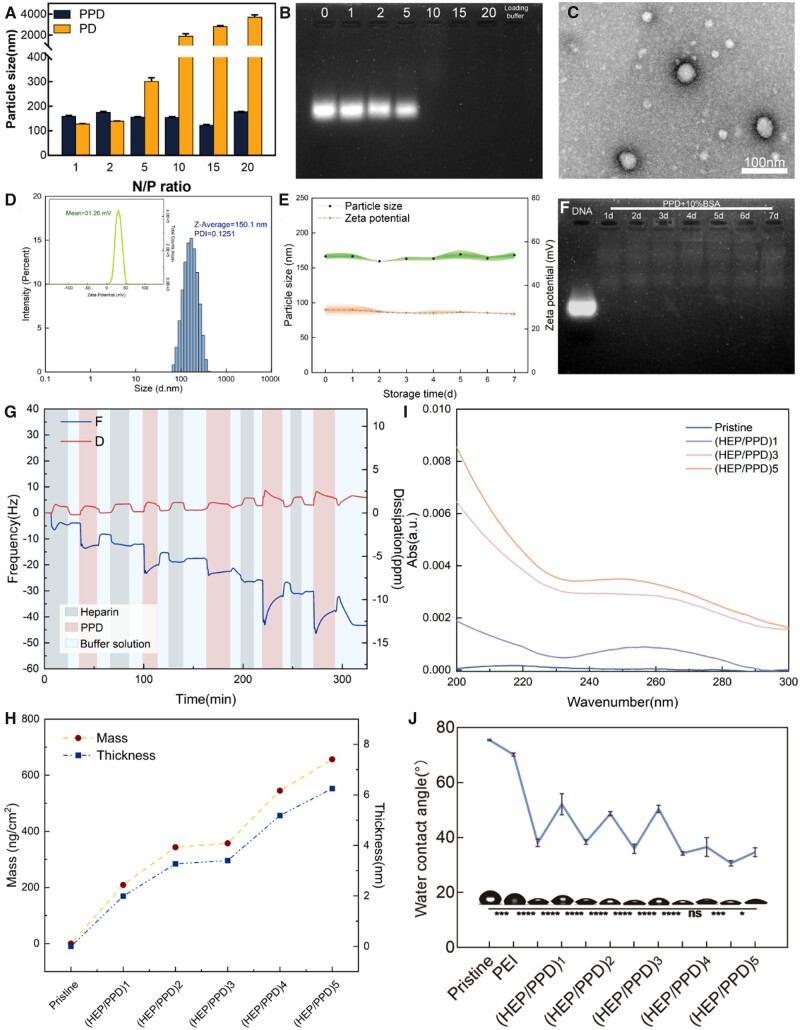
(**A**) The particle size and potential of PD and PPD under different N/P ratios. (**B**) Images of agarose gel electrophoresis under different N/P ratios. (**C**) Particle morphology of gene complexes under TEM (N/P = 10). (**D**) Particle size and zeta potential of PPD under optimal N/P ratio. (**E**) Changes in particle size and zeta potential of PPD for 7 days. (**F**) Nucleic acid leakage from PPD co-incubated with serum for 7 days. (**G**) The total assembly process of (HEP/PPD)5 multilayer membrane characterized by QCM. (**H**) The total assembly process of (HEP/PPD)5 multilayer membrane characterized by UV–vis. (**I**) Variation of multilayer thickness and quality. (**J**) WCA changes during assembly.

As shown in Fig. 1A, the size of the gene complexes is correlated with the N/P ratio and PEG modification [62]. The size of the PEI–g–PEG complex (blue column) was stable at 100–200 nm under each N/P condition, which was convenient for subsequent cellular uptake and nucleic acid delivery. The particle size of the PEI complex (yellow column) increases with the N/P ratio and remains nanoscale when N/P < 10, while it is micron-sized when N/P ≥ 10, which is not suitable for cellular uptake and subsequent gene delivery.

The ability of PEI–g–PEG to bind nucleic acid fragments was assessed by agarose gel electrophoresis [63]. As shown in Fig. 1B, the brightness of nucleic acid bands gradually decreased with increasing N/P ratio, especially in the case of N/P = 1, the brightness was comparable to that in the condition of no polycationic material present (N/P = 0). After N/P ≥ 10, no brightness appeared, indicating that the nucleic acid fragments were completely wrapped inside PEI–g–PEG at this moment, and could not bind to NA-Red for visualization. Therefore, the subsequent N/P ratio screening should be above 10 to avoid the leakage of nucleic acid fragments.

Based on the above experiments, the shape size and distribution of the gene complex (N/P = 10) were observed. As shown in Fig. 1C, the size of the particles in the dry state is <100 nm and the complexes are microspherical with a more uniform distribution. Meanwhile, Fig. 1D shows that the PPD under the N/P ratio of 10, the hydrated particle size is 150.1 nm and the Zeta potential is +31.26 mV. Both the positive charge and the nanoscale properties facilitate the contact of the subsequent gene complex with the negatively charged cell membrane and cellular endocytosis.

### Gene complex stability assay

Figure 1E shows that the particle size of the PPD (N/P = 10) complex did not change significantly in the range of 150–200 nm for 7 days at 4°C, and also its surface potential remained positive, so PPD has favorable storage stability in aqueous solution at 4°C. Figure 1F shows that during co-incubation with 10% BSA, the gel electrophoresis results showed that no significant leakage of nucleic acid inside the PPD was observed, indicating that it also has great serum stability for the delivery of gene fragments *in vivo*.

### Characterization of LBL self-assembly processes

The LBL assembly process was characterized by QCM [64], UV–vis and WCA [56]. Figure 1G shows that during heparin, PPD and buffer replacement, the detected surface frequency of QCM showed an overall decreasing trend, with the frequency dropping to −45.0308 Hz in five cycles, while the surface dissipation changed less, but still increased, from 0 ppm initially to 3.2003 ppm. Both indicate the adsorption of the corresponding substances on the crystal surface. The mass and thickness of the adsorbed substances on the surface were further calculated, and as shown in Fig. 1H, the mass of the (HEP/PPD)5 multilayer film was 656.6 ng/cm^2^ and the thickness was 6.25 nm after five cycles.

The UV–vis results are shown in Fig. 1I, and the absorbance values at 260 nm increased with the increase of the number of layers of HEP/PPD, which can prove the clustering of nucleic acid substances on the surface of the material, indicating the successful preparation of layer-assembled multilayer membranes. The results of WCA in Fig. 1J show that the surface wettability of the multilayer film changes alternately during the deposition of the heparin solution and PPD solution. The surface WCA value is smaller for the outermost layer of heparin, which is more hydrophilic (smaller WCA value), while the WCA value increases for the outer layer of PPD, which is more hydrophobic. The overall trend of the multilayers is more hydrophilic and there is no significant change in the hydrophilicity of the (HEP/PPD)4 bilayer, indicating that the multilayers have reached stability and confirming the successful LBL deposition of the multilayers.

### Characterization of the release process of gene complexes

**Figure 2.**
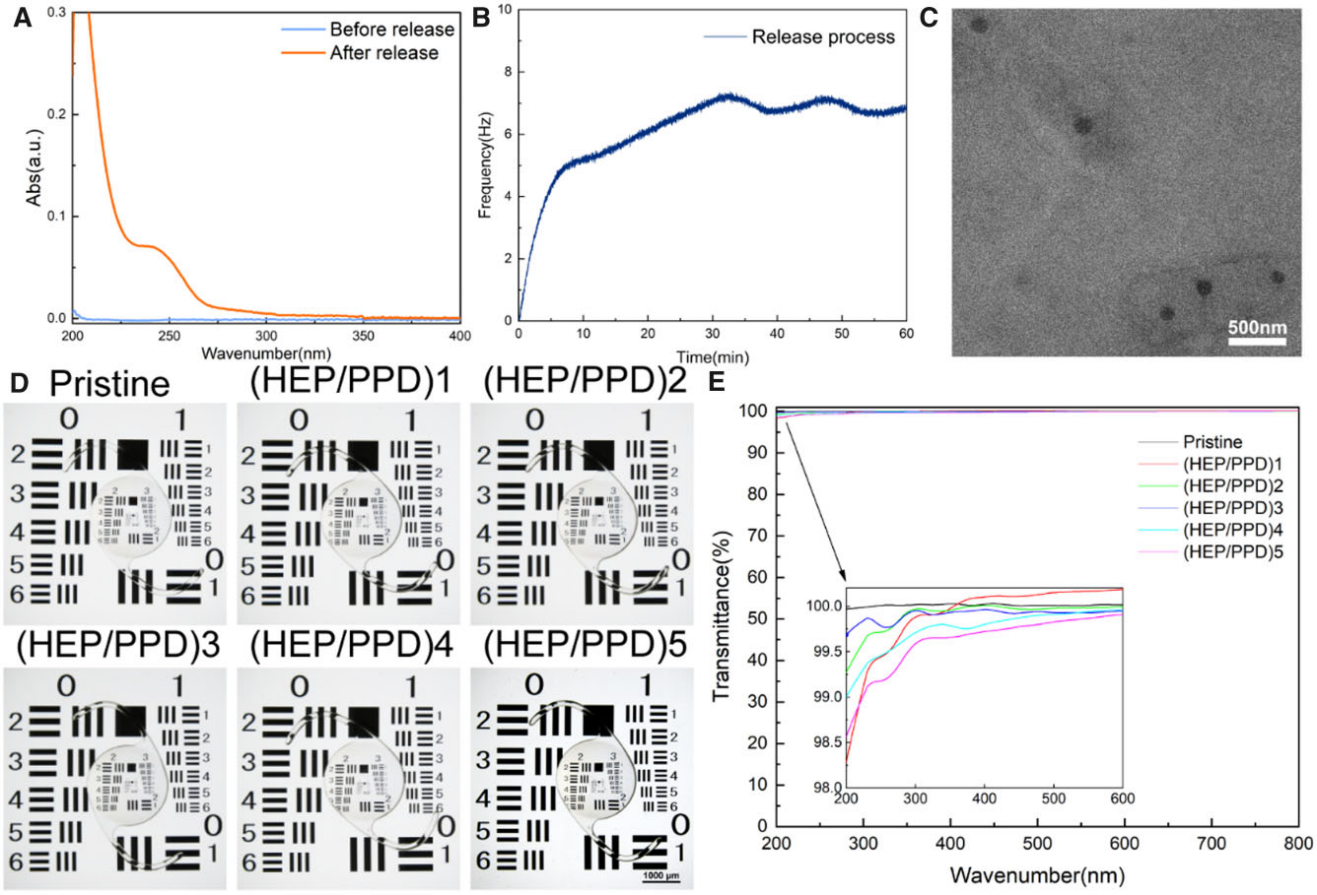
(**A**) Changes of UV absorbance before and after the release of the multilayer film. (**B**) Frequency variation of gene complex release processes in multilayer membranes. (**C**) Particle morphology in release solution.

If the lens is implanted into the eye, the gene complex needs to be successfully released from the multilayer membrane and remain as nano-microspheres in order to successfully deliver nucleic acid fragments to HLECs for subsequent treatment. As shown in Fig. 2A, the absorbance of the release solution before and after immersion in the multilayer membrane was measured by UV–vis. No absorption peak was detected in the pre-release (blue line) liquid, while a significant change in absorption value was observed in the post-release (orange line) liquid, demonstrating that the gene nanoparticles could be successfully shed from the multilayer membrane. Figure 2B shows that during the rinsing of the multilayer film on the surface of the quartz crystal, the frequency increased and then leveled off, indicating that PPD was shed off. The same TEM observation of the collected rinse solution, Fig. 2C shows that the rinse solution contains nanoparticles, which are still granular and of the same size as before, indicating that the gene nanoparticles can be successfully released from the (HEP/PPD)5 multilayer membrane for subsequent cellular uptake and transfection.

### Inspection of optical properties of modified IOLs

Since the prepared (HEP/PPD)5∼IOL serves as a therapeutic IOL, both as a small nucleic acid drug loading platform and as a refractive correction device, it is particularly important to evaluate the optical quality of the IOL before and after modification [57, 64]. Figure 2D shows that imaging of each layer of the modified IOL was performed using a USFA resolution test chart, observed under a stereomicroscope and photographed. The results showed that all layers of (HEP/PPD)1–5∼IOL had no significant changes compared with the unmodified group (Pristine). Similarly, Fig. 2E shows that the transmittance of each modified IOL layer did not change significantly in the visible range (390–780 nm) under UV–vis detection, while a small decrease in transmittance was observed at 260 nm, but all exceeded 98% with good imaging quality.

### *In vitro* biocompatibility evaluation

**Figure 3.**
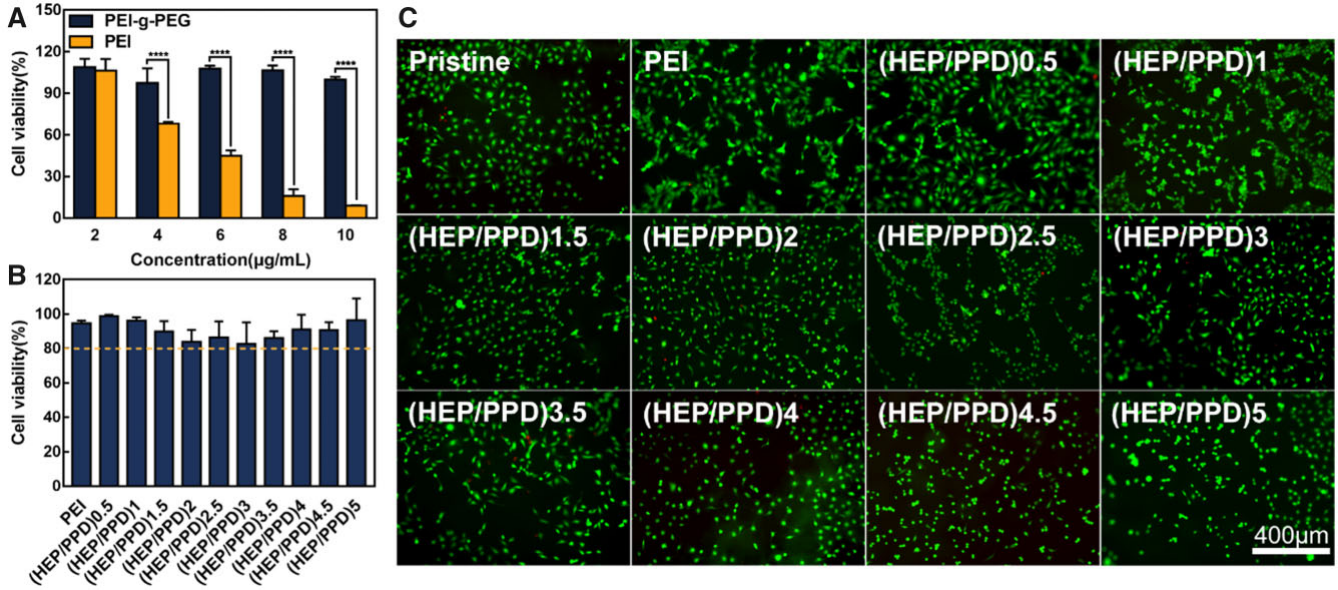
(**A**) Toxicity comparison of PEI and PEI–g–PEG at different concentrations detected by CCK-8. (**B**) Toxicity analysis of CCK-8 on coatings. (**C**) Representative pictures of dead/alive staining on the surface of each coating.

The main evaluation condition for polycationic material as non-viral transfection reagents is whether they are safe and non-toxic to cells [65]. Figure 3A shows the toxicity evaluation of PEI and PEI–g–PEG on HLECs before and after PEG grafting, and it can be found that the cell viability after treatment with each concentration of PEI (orange column) tends to decrease, and the cell viability of HLECs is already below 80% when the PEI concentration is higher than 4 μg/ml. In contrast, the cell viability did not change significantly under the treatment of each concentration of PEI–g–PEG (blue column), which was higher than 90% and had better biocompatibility, indicating that PEI–g–PEG could be used as a gene carrier for disease treatment [66].

In addition, Fig. 3B shows that after modification of heparin and PPD on the surface of PET, the cell viability of HLECs adhering to its surface was higher than 80%, indicating that none of the (HEP/PPD)1–5 multilayers were cytotoxic. Similarly, as shown in Fig. 3C, after dead/alive staining of surface cells, HLECs cells showed green color (Calcein AM staining, live cells), indicating a better cell status, while red fluorescence (PI staining, dead cells) was minimal. The staining results corresponded to the CCK-8 results (Fig. 3B).

### Analysis of cellular uptake capacity

**Figure 4.**
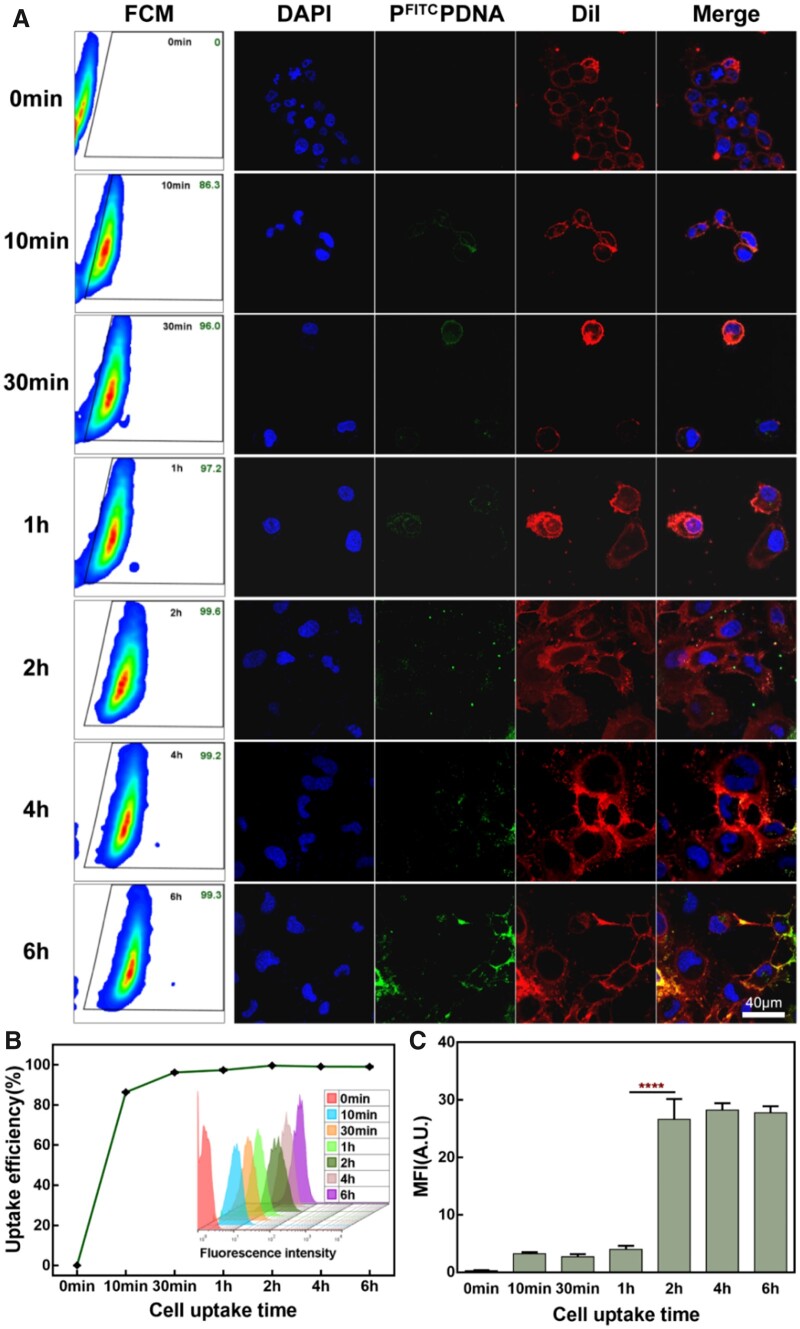
(**A**) HLECs cell uptake of P^FITC^PDNA gene nanoparticles at different times by FCM detection and laser confocal fluorescence channel pictures. (**B**) Statistics of cell uptake FCM results in (A). (**C**) Quantitative statistics of cell uptake fluorescence in (A).

The capacity of endocytosis of gene nanoparticles by HLECs cells directly affects the process by which subsequent nucleic acid fragments perform their corresponding functions [67]. Figure 4A contains FCM results with representative pictures of three fluorescence channels under the microscope. It can be seen that the green fluorescence of the cells gradually increased within 6 h after the addition of P^FITC^PDNA, and the statistics can be obtained in Fig. 4B, 86.3% of the cells had fluorescence signal at 10 min and 99.6% of the HLECs cells could detect green fluorescence after 2 h. This indicates that at 10 min the vast majority of HLEC cells have adsorbed (or taken up) P^FITC^P DNA on their surface. In Fig. 4A, under the microscope, P^FITC^PDNA showed green particles, DiI stained the cell membrane of HLECs red and DAPI stained the nucleus blue. It can be observed that the green fluorescence gradually accumulates on the cell surface (the green signal gradually increases with time), and there is already a green signal inside the cytoplasm of individual cells after 1 h, while many green gene nanoparticles are collected around the nucleus after 6 h. Figure 4C shows the fluorescence quantification of the green signal of the cells. It can be seen that the average fluorescence intensity of the cells increased sharply at 2 h and the fluorescence intensity was basically unchanged in the subsequent time. This means that at 2 h each HLEC cell is saturated with P^FITC^PDNA and no further endocytosis of the complex is taking place.

Both of these results show that the gene nanoparticles were able to adhere to the cell membrane in a short period of time and that the cellular uptake was saturated after 2 h of co-incubation. These indicate that the cells have excellent uptake of the shaped gene nanoparticles, which facilitates the subsequent transfection process.

### Analysis of lysosomal escape capacity

**Figure 5.**
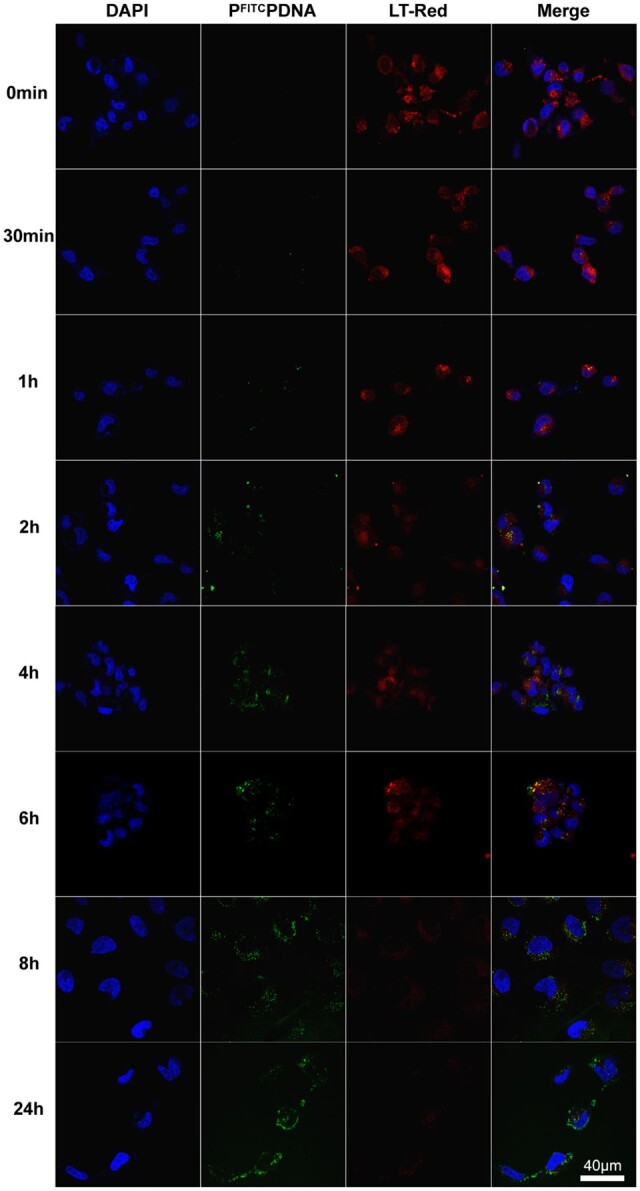
Fluorescence signal recording of P^FITC^PDNA at different time points during a lysosomal escape in HLECs.

Lysosomes are a major obstacle to overcome in gene-drug delivery, and efficient lysosomal escape is a major capability required for gene-delivery vectors [68]. Figure 5 shows that P^FITC^PDNA is green particles, LT-Red stains HLECs lysosomes in red and DAPI stains nuclei in blue. The green signal gradually increases and the red signal gradually decreases with time. The P^FITC^PDNA complex co-localizes with the red-labeled lysosomes at 1 h and appears yellow. After 6 h of incubation, more yellow and green signals appeared, indicating that more P^FITC^PDNA entered the lysosomes. And after 8 h, the red signal as well as the yellow dotted signal largely disappeared, while the green signal became more dispersed and mostly surrounded the nucleus, indicating that all lysosomes containing gene complexes were ruptured due to the lysosomal rupture ability of P^FITC^PDNA and the nanoparticles were released into the cytoplasm [69]. The above results indicate that PEI–g–PEG possesses a good lysosomal escape ability, which helps the subsequent gene fragments to perform their corresponding functions.

### Transfection capability exploration

**Figure 6.**
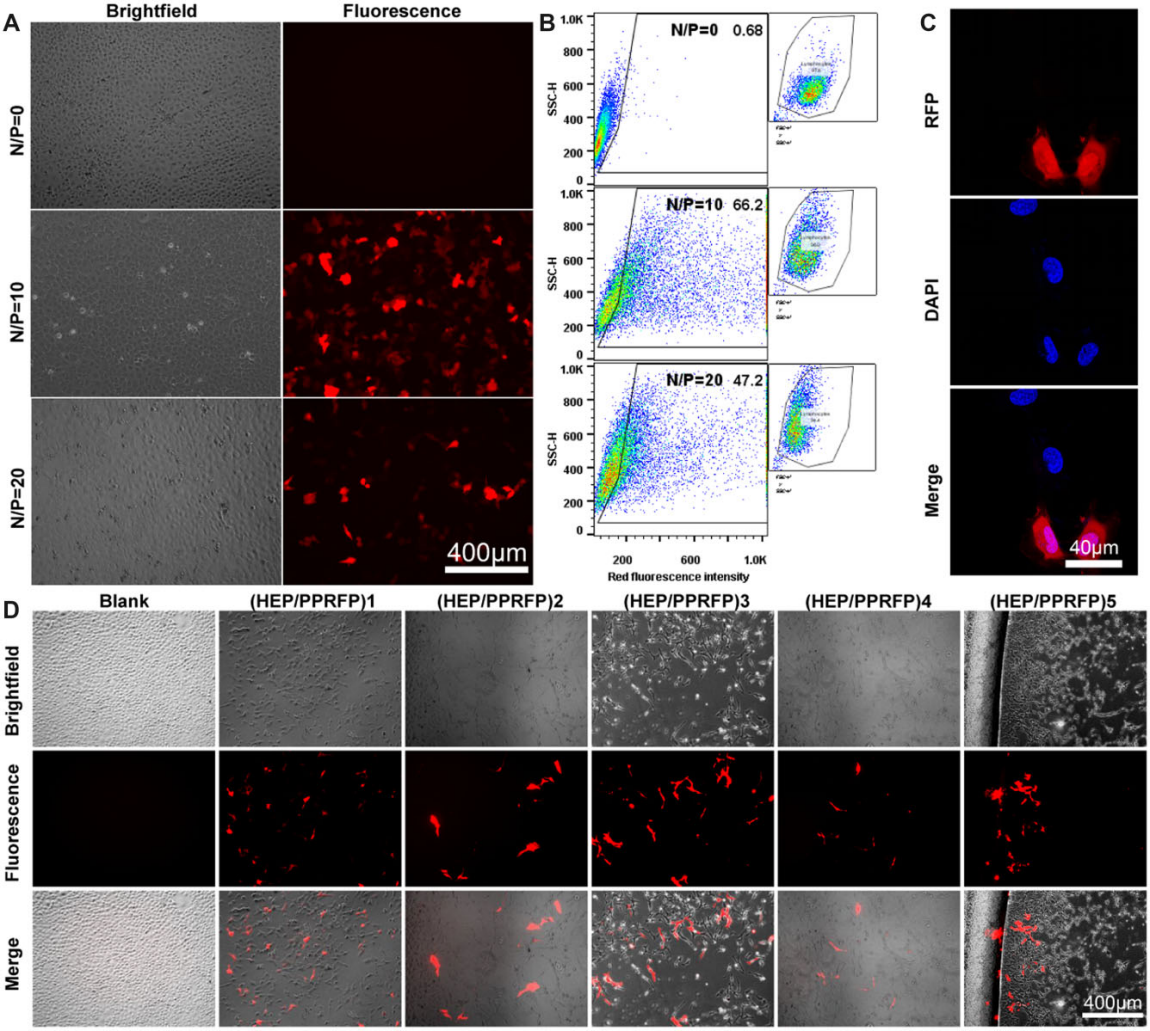
(**A**) The effect of PEI–g–PEG as a gene carrier on the expression of red fluorescent protein under the N/P ratio of 10 and 20. (**B**) FCM results of each N/P group in (A). (**C**) Microstructural observation of red fluorescent protein expression under the N/P ratio of 10 in (A). (**D**) Transfection effect of (HEP/PPRFP)5 layers on HLECs.

HLECs were successfully transfected with the red fluorescent protein under N/P = 10 and 20 conditions shown in Fig. 6A, while the transfection effect of N/P = 20 was weaker than that of N/P = 10. Correspondingly, the FCM statistics in Fig. 6B also showed that the transfection rate of N/P = 10 could reach 66.2%, while the transfection rate of N/P = 20 could reach 47.2%. Therefore, N/P = 10 should be selected as the transfection ratio for subsequent experiments. Figure 6C shows the transfection effect of HLECs on red fluorescent protein at high magnification and the red signal can be seen in the whole cell outline. Figure 6D shows that the material modified with HEP/PPRFP in each layer can successfully transfect HLECs cells in the 24-well plates with a red fluorescent protein.

### IF analysis

**Figure 7.**
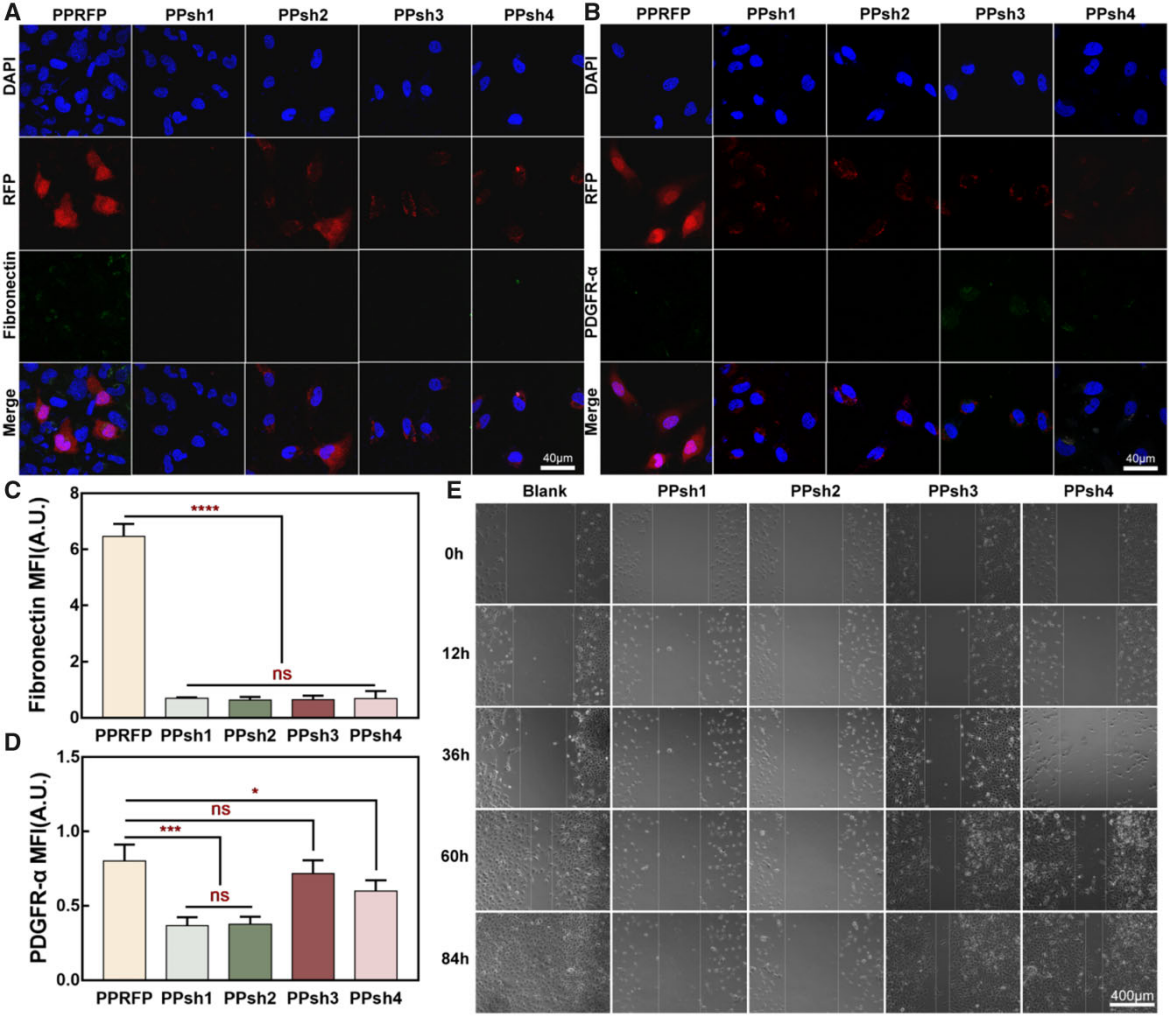
(**A**) IF analysis of EMT-associated protein (fibronectin) in HLECs cells after the treatment of PPsh1–4. (**B**) IF analysis of targeted silencing protein (PDGFR-α) in HLECs cells after the treatment of PPsh1-4. (**C**) Fluorescence quantitative statistics of fibronectin protein in (A). (**D**) Quantitative statistics of PDGFR-α protein fluorescence in (B). (**E**) Characteristic pictures of HLECs cell migration ability analysis after the treatment of PPsh1-4.

Figure 7A and B shows the expression of target protein PDGFR-α and EMT marker protein fibronectin in HLECs cells after treatment with four interfering fragments of PDGFR-α shRNA [12]. It can be seen that the green fluorescent signals representing both proteins were attenuated after PPshn treatment. For quantitative analysis, fluorescence analysis was performed on each group of samples. As shown in Fig. 7C and D, fibronectin protein expression was reduced in both groups, and there was a statistical difference between each treatment group compared with the control group. For PDGFR-α protein expression, it was reduced to different degrees with each group, but the difference between the PPsh1 and PPsh2 groups was more significant compared with the control group, indicating that the interference effect of these two sequences was more effective.

### Analysis of the ability to inhibit cell migration

Since cell migration is the most critical feature of EMT, the scratch experiment was performed to test the effect of four types of PDGFR-α shRNA on the motility activity of HLECs cells. As shown in Fig. 7E, the cells of the blank group grew over the entire scratch area within 84 h of observation, while the migration of HLECs cells was attenuated in all treatment groups, with the anti-migration effect of both PPsh1 and PPsh2 groups being obvious and the effect of PPsh3 and PPsh4 being weaker.

### *In vivo* anti-PCO efficacy and safety evaluation

**Figure 8.**
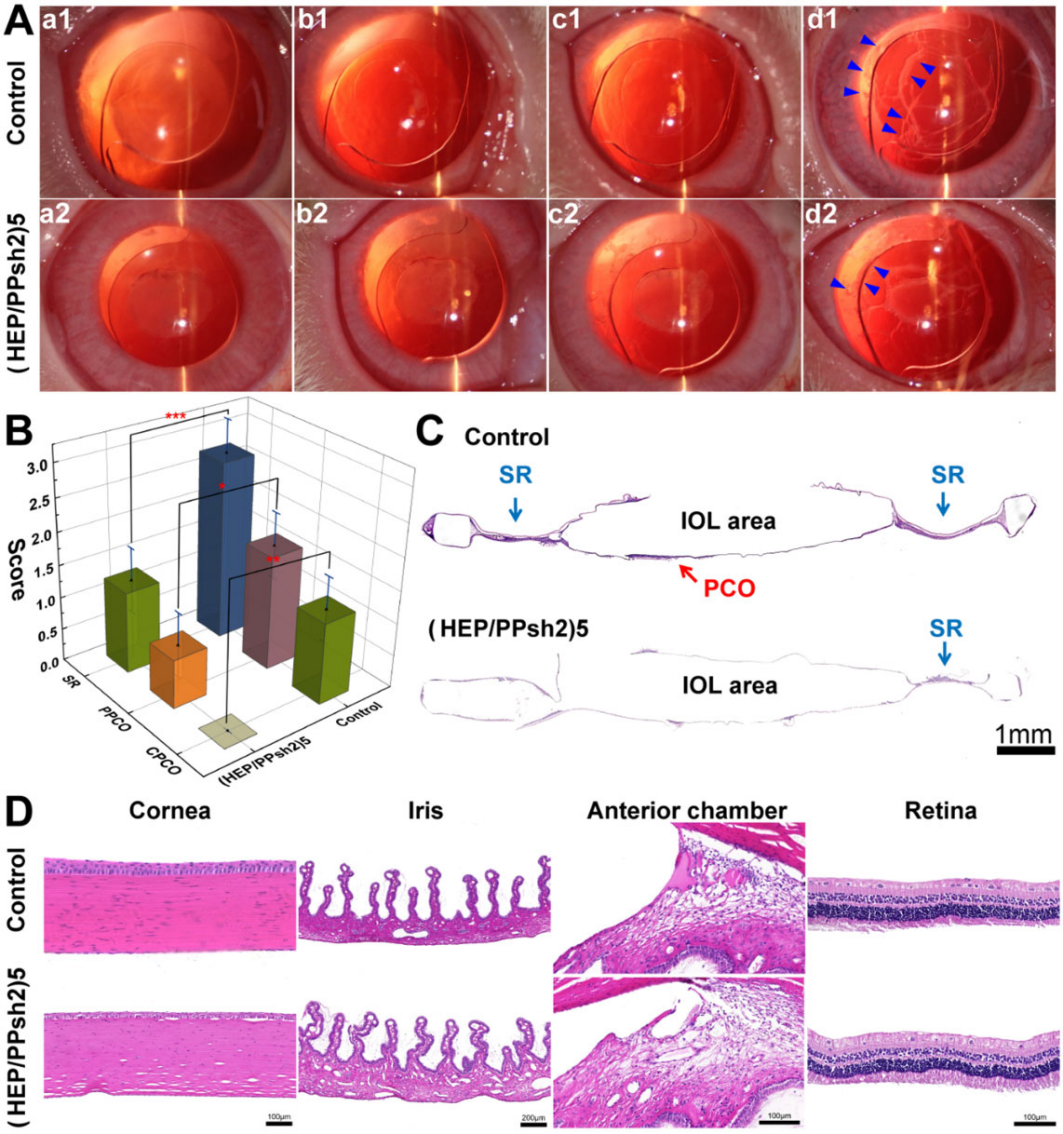
(**A**) Representative slit lamp microscope images at different times after surgery. (**B**) Statistical analysis of PCO scoring. (**C**) HE staining images of capsules of (HEP/PPsh2)5∼IOL group and unmodified control group. (**D**) Representative HE staining images of cornea, iris, anterior chamber angle and retina in the (HEP/PPsh2)5∼IOL group and the unmodified control group.

Combining the results of the previous experiments, the N/P ratio of 10 was chosen for the encapsulation of PDGFR-α shRNA2 with IOL multilayer modification. The development of postoperative PCO could be observed slit lamp observation in 1 day, 3 days, 1 week and 2 weeks postoperatively (Fig. 8A, a–d, respectively). The blue arrow in the follow-up observation, the contraction and thickening of the capsule opening and the presence of numerous folds in both groups were observed from the location of slit. The blue arrow (Fig. 8A, d1 and d2) indicates that the proliferating cells on the surface of the IOL lay flat in a granular shape and appear grayish-white when illuminated by a slit lamp. Most of the cell proliferation in the control group was located in the part of the IOL optical area, while (HEP/PPsh2)5∼IOL was mostly located outside the optical area.

To assess the development of PCO and the effect of the IOL on light transmittance, PCO was divided into Soemmering ring (SR), peripheral PCO (PPCO, hyperplasia in the peripheral area of the IOL), and central PCO (CPCO, hyperplasia in the central area of the IOL). Scores range from 0 to 3, depending on the severity of opacity. A score of 0 indicates no visible PCO and a score of 3 indicates severe opacities. The entire capsules were divided into four quadrants and the severity of the SR, CPCO and PPCO was scored and averaged. As shown in Fig. 8B, the capsule opacity of each part of the lens is calculated using a stereoscope. The values of each part in the control group were higher than those in the (HEP/PPsh2)5∼IOL group, and the difference was statistically significant. The degree of opacity in each group gradually became clearer from the peripheral part to the central optical area, and the CPCO score was 0 in the (HEP/PPsh2)5∼IOL group, while it was 1.25 ± 0.5 in the control group.

To further understand the severity of PCO, pathological sections and HE staining were performed on both groups of capsules to observe the extent of proliferation of HLECs in the capsule. As shown in Fig. 8C, the control group showed a large amount of proliferation in the central posterior capsule and the cells could seriously affect the patient’s postoperative vision. In addition, the proliferation was severe in the equatorial region (SR). In contrast, the (HEP/PPsh2)5∼IOL group had significantly fewer cells than the control group and produced fewer cellular fibrils. The above results suggest that (HEP/PPsh2)5-modified IOL may reduce the severity of PCO to some extent and inhibit the occurrence of PCO.

The pathological tissue sections were used to observe whether the modified IOL implantation caused any effect on the structure and morphology of the periocular tissues. As shown in Fig. 8D, there were no significant differences in the morphology and structure of the cornea, iris, anterior chamber angle and retina among the groups. The corneas of all groups maintained an intact five-layer structure with a tight interlayer arrangement. The iris sections could identify the large vascular structures and small villi-like structures. The trabecular meshwork of the anterior chamber angle was intact and the retinal structures of all groups were intact without obvious disorders. The above results confirmed that (HEP/PPsh2)5-modified IOL has favorable biocompatibility and safety after implantation.

## Conclusion

In summary, this study achieved efficient shRNA delivery in HLECs cells with the help of PEI–g–PEG polycationic material. In addition, the (HEP/PPsh2)5 multilayer membrane was successfully loaded on the surface of IOL using LBL technology. The gene complex formed by PEI–g–PEG was shown to form nanoscale particles with great storage stability and serum stability. The measurements of QCM, UV–vis and WCA showed that (HEP/PPD)5∼IOL was successfully constructed and still maintained excellent optical properties while demonstrating that nanospheres could still be released from the multilayer membrane. *In vitro* cellular experiments showed that PEG modification significantly reduced cytotoxicity, while (HEP/PPD)5∼IOL still possessed excellent cytocompatibility. (HEP/PPRFP)5 showed favorable cellular uptake and lysosomal escape ability, and all layers of (HEP/PPRFP)5 could be successfully transfected with the corresponding encoded proteins. The PDGFR-α shRNA plasmid was encapsulated with the ability to interfere with EMT and anti-cell migration. *In vivo* animal experiments also showed that (HEP/PPsh2)5∼IOL possesses certain PCO prevention effects and excellent intraocular biocompatibility. Thus, PEI–g–PEG provides a reliable platform for the development of efficient shRNA-based gene therapy through a simple electrostatic encapsulation method. Meanwhile, this work will provide a different idea for small nucleic acid drug coatings modified on the surface of IOLs.
